# Efficacy of Acupuncture Treatment for Incidence of Poststroke Comorbidities: A Systematic Review and Meta-Analysis of Nationalized Cohort Studies

**DOI:** 10.1155/2022/3919866

**Published:** 2022-02-01

**Authors:** Li-Kung Wu, Chung-Shan Hung, Yen-Lun Kung, Zhong-Kui Chen, Shinn-Zong Lin, Jaung-Geng Lin, Tsung-Jung Ho

**Affiliations:** ^1^Department of Chinese Medicine, Hualien Tzu Chi Hospital, Buddhist Tzu Chi Medical Foundation, Hualien, Taiwan; ^2^School of Post-Baccalaureate Chinese Medicine, Tzu Chi University, Hualien, Taiwan; ^3^Department of Aging and Community Health, Hualien Tzu Chi Hospital, Buddhist Tzu Chi Medical Foundation, Hualien, Taiwan; ^4^Department of Public Health, Tzu Chi University, Hualien, Taiwan; ^5^Integration Center of Traditional Chinese and Modern Medicine, Hualien Tzu Chi Hospital, Hualien, Taiwan; ^6^Bioinnovation Center, Tzu Chi Foundation, Department of Neurosurgery, Hualien Tzu Chi Hospital, Buddhist Tzu Chi Medical Foundation, Tzu Chi University, Hualien, Taiwan; ^7^Department of Neurosurgery, Hualien Tzu Chi Hospital, Buddhist Tzu Chi Medical Foundation, Hualien, Taiwan; ^8^Research Center for Chinese Medicine & Acupuncture, China Medical University, Taichung, Taiwan; ^9^School of Chinese Medicine, China Medical University, Taichung, Taiwan

## Abstract

Acupuncture has been applied as a complementary therapy in stroke survivors worldwide and approved to be beneficial to stroke recovery. However, there is little medical evidence regarding the association between acupuncture and the risk of poststroke comorbidities. We reviewed big data studies from the Taiwan National Health Insurance Research Database to investigate the risk of poststroke comorbidities after acupuncture treatment in a real-world situation. Ten English (PubMed, Embase, Medline, Cochrane, Alt HealthWatch, CINAHL, Health Source, PsycINFO, PsycARTICLES, and Psychology and Behavioral Sciences Collection) and two Chinese (AiritiLibray and Visualizing Health Data) electronic databases were searched from inception until December 2020 for nationalized cohort studies comparing the effects of acupuncture treatment with a nonacupuncture control group among stroke patients. Eight nationalized cohort studies were included. Six of eight studies showed a moderate overall risk of bias, while two studies showed a serious overall risk of bias. Included studies have investigated the effect of acupuncture in reducing the risk of seven medical conditions after stroke, including stroke recurrence, new-onset acute myocardial infarction (AMI), pneumonia, dementia, epilepsy, urinary tract infection (UTI), and depression. The meta-analysis showed clinically significant reductions in the risk of poststroke comorbidities in the acupuncture group compared to the nonacupuncture group (HR, 0.776; 95% CI, 0.719–0.838; *p* < 0.0001). In this systematic review and meta-analysis of nationalized cohort studies, acupuncture showed clinically relevant benefits in reducing the incidence of poststroke comorbidities, such as stroke recurrence, new-onset acute myocardial infarction (AMI), pneumonia, dementia, epilepsy, and UTI.

## 1. Introduction

Stroke is the second leading cause of death and a major cause of acquired disability worldwide [1]. Among stroke patients, acupuncture has been practiced in traditional Chinese medicine for thousands of years and applied as a complementary therapy in stroke survivors [2]. Acupuncture has been authenticated to improve motor impairment [3], alleviate neurological deficiency [4], reduce psychological symptoms [5], increase local blood circulation [6], and modulate immunology [7] for the stroke patient. Nevertheless, comorbidities and complications of stroke have a huge impact on the prognosis of stroke [8, 9]. Stroke patients display a higher susceptibility to neurological disorders (such as seizure, epilepsy, dementia, and cognitive impairment), infectious diseases (pneumonia and urinary tract infection, UTI), cardiovascular diseases (deep venous thrombosis and acute myocardial infarction, AMI), psychological disorders (depression), and secondary stroke [10]. However, there is little medical evidence regarding the association between acupuncture and the risk of poststroke comorbidities.

Under National Health Insurance coverage, people in Taiwan can receive 15 acupuncture treatments per month in a hospital or at a clinic after stroke. With inexpensive cost and without obvious adverse events [11], acupuncture in stroke patients is well accepted and gradually increasing in Taiwan [2].

Although well-designed randomized control trials (RCT) have proved the efficacy of acupuncture for stroke recovery [12], the limitations, such as small sample size, difficulty in blinding of acupuncture, risks of bias due to deviations from intended interventions, and a lack of long-term follow-up, may negatively influence the reliability and generalization of the outcome.

Moreover, the rigorous-designed clinical trial differs from real-world situations. In clinical practice, acupuncture was conducted by traditional Chinese doctors with diversified and individualized manipulation and acupoint selection. Additionally, in a busy clinical setting, it may be impossible for traditional Chinese doctors to follow the rigorous protocols of RCTs. Traditional Chinese doctors seldom treat specific diagnoses or symptoms but provide holistic intervention that considers the balance of the whole person. Despite the same symptoms, traditional Chinese medical treatment may differ between patients with different physical constitutions. Therefore, the well-established protocol of RCT is not applicable to clinical traditional Chinese medicine practice.

However, investigation of the effect of clinical traditional Chinese treatment is indispensable for both doctors and patients. The cohort study is the best study design that can provide evidence-based information of real-world clinical practice with a long-term prognosis and a large population. In Taiwan, the Taiwan National Health Insurance Research Database (NHIRD) provides anonymous medical records of all insured individuals and was used by numerous cohort studies to reveal clinical practice behavior. To investigate the risk of poststroke comorbidities after acupuncture treatment in a real-world situation, we conducted a systematic review of nationwide cohort studies that have used big data from the NHIRD.

## 2. Methods

### 2.1. Search Strategy

**Figure 1:**
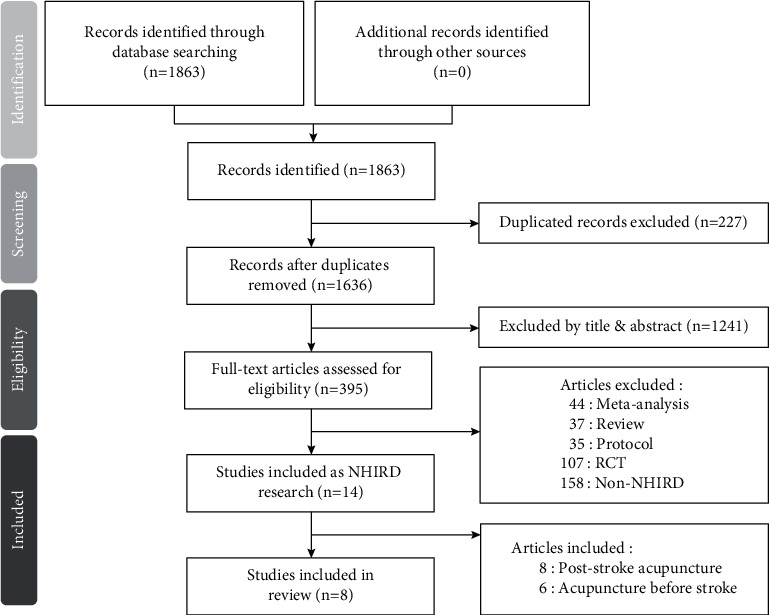
Preferred reporting items for systematic reviews and meta-analyses (PRISMA) guidelines indicating parameters for the search and identification of included studies.

Ten English electronic databases (PubMed, Embase, Medline, Cochrane, Alt HealthWatch, CINAHL, Health Source, PsycINFO, PsycARTICLES, and Psychology and Behavioral Sciences Collection) and two Chinese electronic databases (AiritiLibray and Visualizing Health Data) were searched from inception until December 2020. Key search queries were (Stroke OR Cerebrovascular disorder OR Cerebrovascular Accident OR cerebrovascular disease OR cerebral ischemia OR ischemic stroke OR hemorrhagic stroke OR brain infarction OR cerebral infarction OR CVA) AND (Acupuncture OR Electroacupuncture OR electro-acupuncture OR Auricular Acupuncture OR Ear Acupuncture OR Scalp Acupuncture OR Acupuncture Point OR Acupoint) AND (NHIRD OR National Health OR Health Insurance OR Nation^*∗*^). Reference lists of full-text papers were searched. Relevant studies were also screened.  Figure 1 shows the systematic literature search performed by two authors (Wu and Hung).

### 2.2. Inclusion and Exclusion Criteria

Included studies met the following criteria. (1) Taiwan's National Health Insurance Research Database, (2) participants of the study were diagnosed with stroke by the International Classification of Diseases, 9th Revision, Clinical Modification codes (ICD-9-CM 430–437), (3) the study compared acupuncture with nonacupuncture control groups, and (4) the study showed changes in poststroke comorbidities or medical outcome. Other interventions, such as traditional Chinese medicine, laser acupuncture, acupressure, and massage, were excluded. Studies that did not evaluate acupuncture for stroke-related medical outcomes in NHIRD were excluded.

### 2.3. Data Abstraction and Rating of Articles for ROB

Study selection, data extraction, risk of bias assessment, and quality evaluation were performed independently by two authors (Wu and Hung). The condition, trial sample size, study duration, outcome incidence, between-group hazard ratio, and log-rank test were extracted from selected studies. Two authors rated articles for the confounding, selection of participants, classification of interventions, deviations from intended interventions, missing data, measurement of outcomes, selection of the reported result, and overall bias via the assessment tool for ROBINS-I [13] from the Cochrane Handbook for Systematic Reviews of Interventions [14, 15]. Rating differences were settled by discussion or by a third person (Ho) if consensus was not achieved.

### 2.4. Data Analysis

Data were summarized using hazard ratios (HR) with 95% CIs for binary outcomes or mean difference (MD) with a 95% CI for continuous outcomes. We used R [16] and the package meta [17] for meta-analysis if the trials had acceptable homogeneity of study design, participants, interventions, controls, and outcome measures. Statistical heterogeneity was tested by examining *I*^*2*^ or *p*-value; an *I*^2^ >  50% or a *p*-value < 0.1 indicates the possibility of statistical heterogeneity [18]. Both a fixed-effect model and a random-effects model were used if there was a possibility of statistical heterogeneity among the trials. If the *I*^2^ was <50% or the *p*-value was >0.1, only a fixed-effect model was used for meta-analysis.

## 3. Results

### 3.1. Study Search and Characteristics

Our search strategy retrieved 1,863 potentially eligible articles (Figure 1). A total of 227 duplicates were eliminated. Additional records (1,241) were also excluded because their titles and abstracts were not related to acupuncture, stroke, or Taiwan's NHIRD. After an assessment of full-text articles, 381 records were excluded because of the irrelevance of NHIRD and different study designs. Since the present review emphasized poststroke medical conditions, 6 articles were excluded because the acupuncture treatments were used before the cerebrovascular accident. Eventually, 8 articles were included in our systematic review.

### 3.2. Quality Assessment

**Figure 2:**
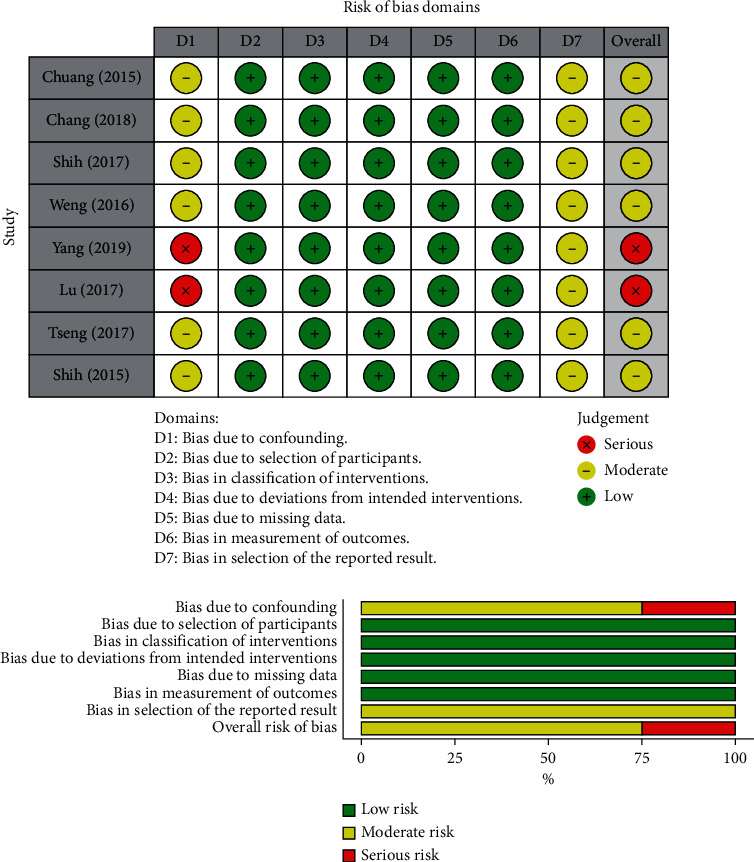
Cochrane risk of bias in nonrandomized studies of interventions (ROBINS-I) for included studies.

The risk of bias of all included studies was assessed using ROBINS-I [13]. Six of eight studies were rated with moderate bias risk due to limitations in study design regarding confounder control (Figure 2). The NHIRD could not control the confounders, such as BMI, the severity of the stroke, family history, education level, cognitive function, mobility function, and so on. However, the six studies have controlled all confounders that could be controlled. Two studies were rated with a serious risk of confounding bias due to uncontrolled known confounders. Yang et al. [19] investigated the risk of UTI without controlling the usage of a bladder catheter. Lu et al. [20] investigated the incidence of depression after acupuncture without controlling the acupuncture sections after the acute stage. Owing to the nature of NHIRD studies that analyze the records from a big database, the risks of classification, deviations from intended interventions, missing data, and the measurement of outcomes were low in all studies. The outcomes of the NHIRD were the risk or incidence of specific diagnosis after the intervention. Therefore, the risk of bias in outcome measurements was low in NHIRD studies. The risk of bias in the selection of the reported results was moderate in all studies because there is no pre-registered system for the NHIRD or big data studies. To summarize, six of eight studies were categorized with a moderate overall risk of bias due to the limitation of study design in confounder control and selection of the reported results [21–26]. Two studies were categorized with a serious overall risk of bias due to failure to control the known and controllable confounders [19, 20].

### 3.3. Outcomes

Studies have investigated the effect of acupuncture in reducing the risk of seven medical conditions after stroke, including six studies involving stroke recurrence, new-onset AMI, pneumonia, dementia, epilepsy, and UTI, and two studies involving poststroke depression (Table 1). Compared to stroke patients who did not receive acupuncture, stroke patients who had received acupuncture displayed a lower incidence of stroke recurrence in ischemic stroke (HR: 0.88; 95% CI: 0.84–0.91), lower incidences of new-onset AMI (HR: 0.86; 95% CI: 0.80–0.93), pneumonia (HR: 0.86; 95% CI: 0.82–0.90), dementia (HR: 0.73; 95% CI: 0.66–0.80), epilepsy (HR: 0.74; 95% CI: 0.68–0.80), and UTI (HR: 0.76; 95% CI: 0.73–0.80).

The association of acupuncture and poststroke depression was inconsistent in the two studies. Tseng et al. indicated the reduced risk of poststroke depression with HR of 0.48 (95% CI: 0.39–0.58) and 0.72 (95% CI: 0.61–0.84) for patients who received frequent and infrequent acupuncture treatments, respectively. Conversely, Lu et al. reported an HR of 1.04 (95% CI: 0.84–1.29) The meta-analysis result of the two studies was provided in the Supplemental Figure S1, the result showed a HR of 0.70 (95% CI: 0.47-1.05) favors acupuncture group but without significant difference.. However, it should be noted that Lu et al. defined stroke patients who received any acupuncture therapies within three months of the discharge as acupuncture users. This definition of acupuncture users may include patients who did not receive enough dosage of acupuncture intervention for observable changes, may exclude patients who received acupuncture after three months of discharge, and may result in an underestimation of the effects of acupuncture treatment.

**Figure 3:**
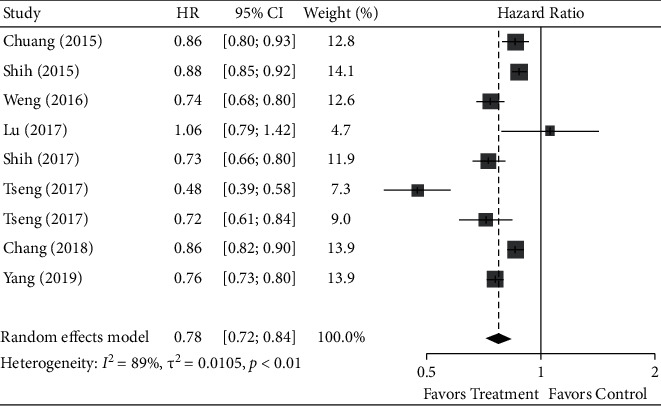
Forest plot of hazard ratio (HR) of poststroke comorbidities with acupuncture intervention compared with nonacupuncture control.

The pooling analysis of eight trials showed that acupuncture treatment significantly reduced the risk of poststroke comorbidities compared to the nonacupuncture group in a random-effects model (HR, 0.776; 95% CI, 0.719–0.838; *p* < 0.0001) with significant heterogeneity (*I*^*2*^ = 89.2%; 95% CI, 81.7%–93.6%). The forest plot of meta-analysis results is shown in Figure 3 (Supporting data for forest plot are provided in the Supplemental Table S1). The results from Tseng et al. were separated into two trials (frequent and infrequent acupuncture users compared with acupuncture nonusers) in the pooling analysis.

### 3.4. Subgroup Analysis

The extraction data of subgroup analysis are shown in Table 2. The effects of acupuncture on acute myocardial infarction, pneumonia, dementia, epilepsy, UTI, and stroke recurrence risk were significant in both genders, with a tendency toward favoring the female gender [19, 21, 23–26]. However, the results are controversial regarding poststroke depression [20, 25].

In the subgroup analysis of different types of strokes, our review indicated the positive influence of acupuncture therapy in reducing the risk of pneumonia, epilepsy, and UTI in patients with all stroke subtypes, including hemorrhagic, ischemic, and other stroke types [19, 21, 26].

In the subgroup analysis of the number of medical conditions, patients with more medical conditions had higher hazard ratios for poststroke pneumonia, dementia, UTI, depression, and stroke recurrence [19, 21, 23–25].

**Figure 4:**
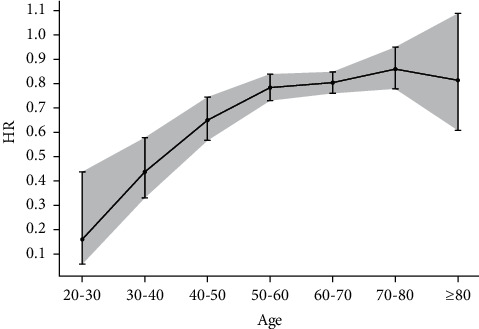
Meta-analysis results showing HRs with 95% confidence intervals of different age strata on poststroke comorbidities after acupuncture treatment.

In different age strata, the younger population displayed lower HRs after acupuncture treatment for poststroke pneumonia, epilepsy, and stroke recurrence. In the meta-analysis of six included studies with age strata [19, 21–24, 26] (Supplemental Table S2), acupuncture treatment showed an age-dependent increase in HR in overall comorbidities after stroke (Figure 4) (Supplemental Table S3).

**Figure 5:**
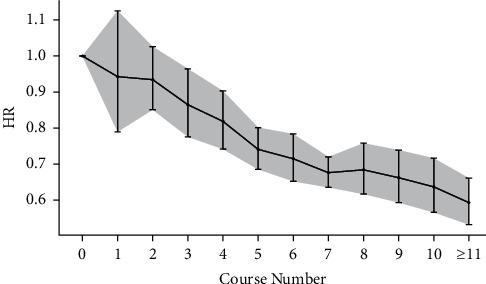
Meta-analysis results showing HRs with 95% confidence intervals of various acupuncture courses on poststroke comorbidities after acupuncture treatment.

The number of acupuncture treatments also plays a crucial role in the incidence of poststroke comorbidities. The risk of acute myocardial infarction [22], pneumonia [21], epilepsy [26], UTI [19], and stroke recurrence [23] exhibited a dose-dependent decrease with increasing sessions of acupuncture treatment (Supplemental Table S4). The meta-analysis of the above five included studies showed a lower risk for overall poststroke comorbidities as the number of acupuncture treatment packages increased (Figure 5) (Supplemental Table S5). Tseng et al. showed the probability of poststroke depression in patients was reduced with frequent acupuncture (HR: 0.475 (0.389–0.580)) and infrequent acupuncture (HR 0.718 (0.612–0.842)), compared to those not receiving acupuncture treatments [25].

To summarize, the effects of acupuncture treatment on poststroke comorbidities are associated with gender, the number of medical conditions, age, and the number of acupuncture treatments received.

## 4. Discussion

Regarding the limitations of RCT that cannot reflect the real effect of clinical practice, this review identified eight NHIRD studies involving acupuncture for poststroke comorbidities. The results indicated that patients who had received acupuncture were at lower risk of poststroke comorbidities, including AMI, pneumonia, dementia, epilepsy, UTI, and stroke recurrence. To our knowledge, the review is the first study to summarize the results of big data analyses from NHIRD. NHIRD studies with nationalized records of acupuncture that cumulated from real-world practice can provide the most direct evidence for clinical use without excluding the diversity and individualization of the traditional Chinese intervention from study procedures.

### 4.1. Evidence-Based Medicine for Acupuncture in the Treatment of Stroke Patients

Many empirical studies investigated the effects of acupuncture on comorbidities after stroke. In studies related to poststroke pneumonia, acupuncture is effective in decreasing the incidence and morbidity of pneumonia in patients with poststroke syndrome [27, 28]. For urology, acupuncture was an effective treatment for poststroke urodynamic detrusor overactivity [29]. Electroacupuncture also showed beneficial effects on stroke survivors with incomplete bladder emptying, thus improving urinary function [30]. Besides, acupuncture showed a clinically relevant decrease in stroke relapse compared with sham acupuncture [31]. For poststroke insomnia, a meta-analysis showed that acupuncture appeared to be more effective than conventional drugs [32]. For vascular dementia, scalp acupuncture could improve the clinical intelligence level of dementia induced by cerebral infarction [33]. For poststroke depression, filiform needle acupuncture can reduce the Hamilton Depression Rating Scale (HAMD) level compared to anti-depressant drugs after treatment for two weeks [5]. Another systematic review and meta-analysis also indicated that acupuncture shows greater effects on poststroke depression, with a better safety profile than anti-depressants [34]. Overall, for poststroke comorbidities, acupuncture showed positive effects on pneumonia, urinary function, stroke relapse, insomnia, vascular dementia, and depression in stroke survivors in rigorous-designed experimental environments.

In addition to the effects on poststroke comorbidities, acupuncture has proven effects on symptoms, sequelae, and functions in a well-controlled experimental environment regarding evidence of RCT, systematic review, and meta-analysis [35, 36]. Acupuncture effectively reduces neurological impairments [11, 37], motor impairments [3, 38–40], dysphagia [37, 41], and other symptoms, such as fatigue [42], insomnia [32], and pain [43]. Acupuncture also assists with cognitive function [4] and activities of daily living [3, 39] after stroke. Moreover, acupuncture increases mean flow velocity in both hemispheres [6], displays protective effects on brain reperfusion injury [44], and improves neurogenesis [45].

### 4.2. Mechanism of Acupuncture for Poststroke Comorbidities

The mechanism behind how acupuncture reduces the incidence of poststroke comorbidities is still unclear. However, there are several possible underlying mechanisms, including immunological, neurological, hemodynamic, neuroendocrine, and metabolic mechanisms.

Regarding the immunological mechanism, acupuncture can directly modulate specific and nonspecific immunity to correct systemic immunosuppression and decrease susceptibility to infection after stroke [46]. Acupuncture decreases certain cytokines and inhibits Th1 cell responses to regulate Th1/Th2 balance [47, 48], which circumvent infectious diseases [49]. Furthermore, acupuncture is related to systemic anti-inflammatory responses, which are mediated by reducing endotoxin-induced inflammation [50], decreasing whole blood tumor necrosis factor-*α* (TNF-*α*) [51], and upregulating IL-10 production [52]. These studies demonstrate that acupuncture may contribute to modulating immune system balance, reducing the inflammatory response, and correcting systemic immunosuppression and may lead to improvements in poststroke UTI and pneumonia incidences [46, 49].

In the neurological mechanism, acupuncture can stimulate multiple brain networks and regulate functional connectivity by activating under-activated brain regions while deactivating overloaded areas [53], which may relate to how acupuncture alleviates incidences of epilepsy and dementia. Moreover, in terms of the autonomic nerve system, the autonomic shift may play an important role in increased susceptibility to infections after intracerebral hemorrhage. Acupuncture regulates the activities of the autonomic nervous system by enhancing vagal nerve activity and decreasing sympathetic nerve activity [54]. By correcting autonomic shifts, acupuncture may reduce the susceptibility of infections and decrease the incidence of UTI and pneumonia.

Regarding the hemodynamic mechanism, acupuncture can increase cerebral blood flow [6, 55], enhance local blood perfusion [56], promote angiogenesis [57], and lower blood pressure [58]. Previous studies demonstrated that acupuncture increased nitric oxide generation, which could regulate local blood circulation and relieve atherosclerosis development [56]. These hemodynamics effects may contribute to reducing risks of AMI and recurrent stroke [59].

In the neuroendocrine mechanism, acupuncture therapy upregulates the expression of serotonin 1_A_ receptor (5-HT1_A_ receptor) in the cortex, thalamus, hippocampus, and the hypothalamus, which contribute to alleviating the depressive-like behavior [60]. In addition, acupuncture effectively cures chronic pain [61]. Through regulation of the serotonin receptor and pain reduction, acupuncture may alleviate the causes of depression.

In the metabolic mechanism, acupuncture treatment can reduce serum triglycerides, low-density lipoprotein, cholesterol, and total cholesterol levels [62] and is consequently effective in reducing the risk of AMI and recurrent stroke [63].

In addition to the immunological, neurological, hemodynamic, neuroendocrine, and metabolic mechanisms, other indirect factors may also enhance the effects of acupuncture on poststroke comorbidities. For example, a previous study showed that acupuncture significantly decreases residual urine and may decrease the incidence of UTI [64]. In addition, improved motor ability induced by acupuncture [65, 66] may contribute to better physical mobility, more aerobic activities, and improved cardiopulmonary fitness associated with reduced risks of AMI and recurrent stroke [67, 68].

### 4.3. Subgroup Analysis

According to subgroup analysis results, the effects of acupuncture on poststroke comorbidities were associated with gender, the number of medical conditions, age, and the number of acupuncture treatments received. Acupuncture effects were shown to favor females. The possible underlying mechanism may be due to different neural effects between males and females induced by acupuncture, which were proved in the functional magnetic resonance imaging (MRI) study [69].

Through subgroup analysis, we found that the benefits of acupuncture decrease as baseline comorbidities increase and with the older age. Since the elderly and patients with more medical conditions have a poorer physical constitution and organic function, the curative effect of acupuncture is not easily detected. In addition, patients with older age or complicated medical conditions are more susceptible to infections, which may worsen the outcomes of stroke prognosis. Therefore, traditional Chinese doctors may use other treatment methods, such as moxibustion, laser acupuncture, electroacupuncture, and infrared or far-infrared therapy, to help these patients. Moreover, in the theory of traditional Chinese medicine, acupuncture can regulate but not generate the qi of human meridian circulation [70]. With increased age, meridian qi gradually decreases after 40 years of age [71]. Consequently, elderly patients have little qi to be regulated and show a poorer response after acupuncture [71].

The risk of poststroke comorbidities exhibited a dose-dependent decrease as patients received more acupuncture treatments. Although 15 acupuncture treatments per month was the limit imposed by Taiwan's National Health Insurance System, there is no evidence indicating the optimal dosage of acupuncture for stroke patients. In traditional Chinese medicine theory, acupuncture may regulate yin and yang and modulate the body to become balanced [72]. Therefore, more treatments may help stroke patients balance their health and prevent poststroke comorbidities.

### 4.4. Limitations

The systematic review and meta-analysis of nationalized retrospective cohort studies have some limitations due to the database characteristics and study method. First, the studies used retrospective data claimed from NHIRD, which lacked information regarding possible confounding factors, such as disease severity, stroke lesion characteristics, family history, baseline lifestyle, physical status, psychiatric condition, and laboratory examinations. Second, although the accuracy of ICD-9-CM codes in the database has been proved in previous studies [2], the misdiagnosis that violates the validity of ICD cannot be avoided. Third, information on the actual acupuncture points and manipulations applied in treatment was absent. Diversified and individualized acupoints selection without adequately standardized protocols may have affected the accuracy of the estimated treatment effects. Fourth, since our review was based on observational retrospective cohort studies that are nonrandomized studies, we cannot deduce the cause-and-effect relationship between acupuncture and poststroke comorbidities regarding the significant association. Moreover, the level of evidence of retrospective cohort studies is lower than that of randomized controlled trials. Fifth, the population could be overlapped in several studies since they share the same database with the same inclusion criteria and ICD-9 diagnosis code; therefore, the efficacy of meta-analysis might be limited.

## 5. Conclusions

Our systematic review and meta-analysis of nationalized cohort studies revealed that acupuncture is associated with reduced incidence of poststroke comorbidities such as stroke recurrence, new-onset AMI, pneumonia, dementia, epilepsy, and UTI. The meta-analysis results of eight trials showed that acupuncture exerts a significant effect in reducing the risk of poststroke comorbidities compared to the nonacupuncture group with a hazard ratio: 0.776 (95% CI: 0.719–0.838). Regarding subgroup analysis, HR results involving different age strata and a number of acupuncture treatments received also show a significant correlation after meta-analysis. However, eight included nationwide cohort studies were assessed as moderate to high risk of bias. As a result, further studies that evaluate adverse events and include a long-term follow-up should be conducted to determine the efficacy, safety, and side effects of acupuncture for poststroke comorbidities in real-world settings.

## Figures and Tables

**Table 1:** Main characteristics of included studies.

First author (year)	New stroke survivors	Sample size (acupuncture: non acupuncture)	Propensity match ratio	Mean age or year range	Primary diagnosis of stroke	Diagnosis for inclusion	Intervention	Control	Primary outcome	Follow-up until	Incidence (per 1,000 person-years)	Adjusted HR (95% CI)
Chuang (2015) [22]	182,619	23,475:46,950	1 : 2	40–79	01/01/2000–12/31/2004	ICD-9 CM 430–437	≥1 course of acu	Without acu	AMI (ICD-9-CM 410)	12/31/2009	Acu: 9.2 Nonacu: 10.8	0.86 (0.80–0.93)
Chang (2018) [21]	226,699	12,557:12,557	1 : 1	≥20	01/01/2000–12/31/2004	ICD-9 CM 430–437	≥2 courses of acu	Without acu	Pneumonia (ICD-9-CM 480–486)	12/31/2009	Acu: 53.4 Nonacu: 58.9	0.86 (0.82–0.90)
Shih (2017) [24]	226,699	5,610:5,610	1 : 1	≥50	01/01/2000–12/31/2004	ICD-9 CM 430–437	≥5 courses of acu	Without acu	Dementia (ICD-9-CM 290)	12/31/2009	Acu: 26.5 Nonacu: 34.6	0.73 (0.66–0.80)
Weng (2016) [26]	226,699	21,020:21,020	1 : 1	≥20	01/01/2000–12/31/2004	ICD-9 CM 430–438	≥2 courses of acu	Without acu	Epilepsy (ICD-9-CM 345)	12/31/2009	Acu: 9.8 Nonacu: 11.5	0.74 (0.68–0.80)
Yang (2019) [19]	226,699	9,643:9,643	1 : 1	≥30	01/01/2000–12/31/2004	ICD-9 CM 430–437	≥2 treat of acu	Without acu	UTI (ICD-9-CM 599.0)	12/31/2009	Acu: 37.6 Nonacu: 39.4	0.76 (0.73–0.80)
Lu (2017) [20]	16,046	1,714:14,332 1,714:1,714	Non-1 : 1	≥18	01/01/2002–12/31/2012	ICD-9 CM 430–434, 436–437	≥1 treat of acu	Without acu	Depression (ICD-9-CM 296, 309, 311)	12/31/2013	Acu: 11.1 Nonacu: 9.7 Propensity: Acu: 11.1 Nonacu: 10.6	1.04 (0.84–1.29) Propensity: 1.06 (0.79–1.42)
Tseng (2017) [25]	8,487	Freq U: 1,036 Infreq U: 1,053 Non-U: 6,398	Non-	Freq U: 61.28 Infreq U: 61.77 Non-U: 66.21	01/01/2000–12/31/2005	ICD-9 CM 430–434, 436–437	Freq U: ≥6 acu treat Infreq U: 1–5 acu treat	Without acu	Depression (ICD-9-CM 296, 309, 311)	12/31/2007	Freq U: 15.2 Infreq U: 24.0 Non-U: 34.6	Freq U: 0.475 (0.389–0.580) Infreq U: 0.718 (0.612–0.842)
Shih (2015) [23]	30,058	15,029:15,029	1 : 1	≥30	01/01/2000–12/31/2004	ICD-9 CM 430–434	≥1 course of acu	Without acu	Ischemic stroke (ICD-9-CM 430–434)	12/31/2009	Acu: 69.9 Nonacu: 71.4	0.88 (0.84–0.91)

Note: acu = acupuncture, treat = treatments, U = users, freq U = frequent users, infreq *U*=Infrequent users, non-U = nonusers, course = six consecutive acupuncture treatments.

**Table 2:** Risks of poststroke comorbidities associated with acupuncture treatment in patients with stroke stratified by sex, type of stroke, medical conditions, and age.

Author (year)	Gender	Types of stroke	Baseline medical conditions	Age strata
Chuang (2015) [22]	Female HR: 0.85^*∗*^ (0.76–0.95) Male HR: 0.87^*∗*^ (0.80–0.95)	Hemorrhagic HR: 0.62^*∗*^ (0.44–0.88) Ischemic HR: 0.87^*∗*^ (0.79–0.95) Others HR: 0.89 (0.79–1.01)	Nil	40–49 HR: 0.84 (0.62–1.14) 50–59 HR: 0.75^*∗*^ (0.63–0.90) 60–69 HR: 0.85^*∗*^ (0.75–0.95) 70–79 HR: 0.93 (0.83–1.03)
Chang (2018) [21]	Female HR: 0.79^*∗*^ (0.70–0.82) Male HR: 0.92^*∗*^ (0.86–0.98)	Hemorrhagic HR: 0.66^*∗*^ (0.53–0.81) Ischemic HR: 0.86^*∗*^ (0.81–0.92) Others HR: 0.87^*∗*^ (0.80–0.95)	0 HR: 0.49^*∗*^ (0.43–0.57) 1 HR: 0.77^*∗*^ (0.70–0.85) 2 HR: 0.92 (0.84–1.01) 3 HR: 1.10 (1.01–1.19)	20–29 HR: 0.16^*∗*^ (0.04–0.69) 30–39 HR: 0.52^*∗*^ (0.28–0.98) 40–49 HR: 0.73^*∗*^ (0.58–0.93) 50–59 HR: 0.83^*∗*^ (0.72–0.95) 60–69 HR: 0.82^*∗*^ (0.75–0.89) 70–79 HR: 0.88^*∗*^ (0.81–0.95) ≥80 HR: 0.86 (0.73–1.00)
Shih (2017) [24]	Female HR: 0.70^*∗*^ (0.60–0.80) Male HR: 0.75^*∗*^ (0.66–0.85)	Hemorrhagic HR: 0.77 (0.53–1.12) Ischemic HR: 0.74^*∗*^ (0.66–0.84) Others HR: 0.69^*∗*^ (0.57–0.82)	0 HR: 0.55^*∗*^ (0.39–0.77) 1 HR: 0.64^*∗*^ (0.52–0.80) 2 HR: 0.81^*∗*^ (0.68–0.98) ≥3 HR: 0.82^*∗*^ (0.71–0.95)	50–59 HR: 0.64^*∗*^ (0.47–0.86) 60–69 HR: 0.75^*∗*^ (0.64–0.88) 70–79 HR: 0.72^*∗*^ (0.63–0.83) ≥80 HR: 0.54^*∗*^ (0.38–0.76)
Weng (2016) [26]	Female HR: 0.70^*∗*^ (0.61–0.81) Male HR: 0.77^*∗*^ (0.69–0.85)	Hemorrhagic HR: 0.60^*∗*^ (0.50–0.73) Ischemic HR: 0.86^*∗*^ (0.78–0.96) Others HR: 0.62^*∗*^ (0.52–0.74)	Nil	20–29 HR: 0.16^*∗*^ (0.04–0.68) 30–39 HR: 0.39^*∗*^ (0.21–0.73) 40–49 HR: 0.51^*∗*^ (0.39–0.66) 50–59 HR: 0.66^*∗*^ (0.54–0.80) 60–69 HR: 0.79^*∗*^ (0.68–0.91) 70–79 HR: 0.88 (0.76–1.02) ≥80 HR: 0.71 (0.48–1.04)
Yang (2019) [19]	Female HR: 0.73^*∗*^ (0.69–0.78) Male HR: 0.80^*∗*^ (0.75–0.85)	SAH HR: 0.58^*∗*^ (0.50–0.69) Ischemic HR: 0.78^*∗*^ (0.74–0.83) TIA HR: 0.81^*∗*^ (0.72–0.92) Others HR: 0.72^*∗*^ (0.63–0.82)	0 HR: 0.52^*∗*^ (0.47–0.58) 1 HR: 0.74^*∗*^ (0.69–0.80) 2 HR: 0.95 (0.87–1.03) ≥3 HR: 0.91 (0.83–1.01)	30–39 HR: 0.62 (0.34–1.16) 40–49 HR: 0.65^*∗*^ (0.53–0.79) 50–59 HR: 0.82^*∗*^ (0.73–0.93) 60–69 HR: 0.74^*∗*^ (0.69–0.80) ≥70 HR: 0.77^*∗*^ (0.72–0.82)
Lu (2017) [20]	Female HR: 1.30 (0.90–1.86) Male HR: 1.05 (0.76–1.45)	Hemorrhagic HR: 0.70 (0.42–1.16) Ischemic HR: 1.12 (0.88–1.43)	Nil	<65 HR: 1.19 (0.84–1.68) ≥65 HR: 1.07 (0.77–1.50)
Tseng (2017) [25]	Female HR: 0.78 (0.71–0.86) ^*∗*^Compared to male	Hemorrhagic HR: 0.80^*∗*^ (0.68–0.94) Ischemic HR: 0.91 (0.81–1.03) ^*∗*^Compared to other types of stroke	MI HR: 1.30 (1.08–1.56) Cancer HR: 1.42 (1.27–1.60) DM HR: 1.14 (1.03–1.25) CKD HR: 1.52 (1.35–1.72) COPD HR: 1.22 (1.10–1.36) THI HR: 1.63 (1.34–1.99) ^*∗*^Compared to patients without the medical condition	Older HR: 1.045 (1.040–1.050) ^*∗*^Compared to younger
Shih (2015) [23]	Female HR: 0.83^*∗*^ (0.79–0.89) Male HR: 0.91^*∗*^ (0.86–0.95)	Ischemic HR: 0.88^*∗*^ (0.84–0.91)	HTN no HR: 0.67^*∗*^ (0.62–0.74) HTN yes HR: 0.94^*∗*^ (0.90–0.98) HPL no HR: 0.84^*∗*^ (0.81–0.88) HPL yes HR: 0.97 (0.90–1.04) DM no HR: 0.83^*∗*^ (0.79–0.87) DM yes HR: 0.96 (0.90–1.02)	30–39 HR: 0.35^*∗*^ (0.22–0.54) 40–49 HR: 0.61^*∗*^ (0.52–0.71) 50–59 HR: 0.81^*∗*^ (0.74–0.89) 60–69 HR: 0.85^*∗*^ (0.80–0.91) 70–79 HR: 0.99 (0.92–1.06) ≥80 HR: 1.15 (0.98–1.34)

Note: TIA = transient ischemic attack, SAH = subarachnoid hemorrhage, MI = myocardial infarction, CKD = chronic kidney diseases, COPD = chronic obstructive pulmonary disease, THI = traumatic head injury, DM = diabetes mellitus, HTN = hypertension, and HPL = hyperlipidemia. ^∗^Significant difference.

## Data Availability

The data that support the findings of this study are available from the corresponding authors upon reasonable request.

## Supplementary Materials

Supplementary Materials Table S1. Supporting data for Forest plot of Hazard Ratio (HR) of post-stroke comorbidities with acupuncture intervention compared with non-acupuncture control. A: acupuncture group, C: control group. Table S2. Supporting data extracted from each study showing HRs with 95% confidence intervals of different age strata on post-stroke comorbidities after acupuncture treatment. (HR: hazard ratio; LCI: lower confidence intervals; UCI: upper confidence intervals). Table S3. Meta-analysis results of different age strata on post-stroke comorbidities after acupuncture treatment. (k: number of samples; I2: heterogeneity; p: p-value). Table S4. Supporting data extracted from each study showing HRs with 95% confidence intervals of various acupuncture courses on post-stroke comorbidities after acupuncture treatment. Table S5. Supporting data for Meta-analysis results of various acupuncture courses on post-stroke comorbidities after acupuncture treatment. (k: number of samples; I2: heterogeneity; p: p-value). Figure S1. Forest plot of Hazard Ratio (HR) of post-stroke depression with acupuncture intervention compared with non-acupuncture control.
