# Supplementary material for: Efficacy of Acupuncture Treatment for Incidence of Poststroke Comorbidities: A Systematic Review and Meta-Analysis of Nationalized Cohort Studies
Source: Evid Based Complement Alternat Med. 2022 Feb 1;2022:3919866. doi: 10.1155/2022/3919866 (PMC8825287; doi:10.1155/2022/3919866)
Supplement: Supplementary Materials — Table S1. Supporting data for Forest plot of Hazard Ratio (HR) of post-stroke comorbidities with acupuncture intervention compared with non-acupuncture control. A: acupuncture group, C: control group. Table S2. Supporting data extracted from each study showing HRs with 95% confidence intervals of different age strata on post-stroke comorbidities after acupuncture treatment. (HR: hazard ratio; LCI: lower confidence intervals; UCI: upper confidence intervals). Table S3. Meta-analysis results of different age strata on post-stroke comorbidities after acupuncture treatment. (k: number of samples; I2: heterogeneity; p: p-value). Table S4. Supporting data extracted from each study showing HRs with 95% confidence intervals of various acupuncture courses on post-stroke comorbidities after acupuncture treatment. Table S5. Supporting data for Meta-analysis results of various acupuncture courses on post-stroke comorbidities after acupuncture treatment. (k: number of samples; I2: heterogeneity; p: p-value). Figure S1. Forest plot of Hazard Ratio (HR) of post-stroke depression with acupuncture intervention compared with non-acupuncture control. [file 3919866.f1.zip › Supplementary Materials_final.docx]

**Supplementary Materials**

| **Author** | **Year** | **Total.A** | **Total.C** | **Event.A** | **Event.C** | **adjusted HR** | **LCI** | **HCI** |
| --- | --- | --- | --- | --- | --- | --- | --- | --- |
| **Chuang** | **2015** | 23475 | 46950 | 1376 | 3915 | 0.86 | 0.8 | 0.93 |
| **Shih** | **2015** | 15029 | 15029 | 4639 | 5749 | 0.88 | 0.84 | 0.91 |
| **Weng** | **2016** | 21020 | 21020 | 993 | 1382 | 0.74 | 0.68 | 0.8 |
| **Lu** | **2017** | 1714 | 1714 | 100 | 91 | 1.06 | 0.79 | 1.42 |
| **Shih** | **2017** | 5610 | 5610 | 720 | 1025 | 0.73 | 0.66 | 0.8 |
| **Tseng** | **2017** | 1036 | 6398 | 110 | 1551 | 0.475 | 0.389 | 0.58 |
| **Tseng** | **2017** | 1053 | 6398 | 117 | 1551 | 0.718 | 0.612 | 0.842 |
| **Chang** | **2018** | 12557 | 12557 | 2971 | 3825 | 0.86 | 0.82 | 0.9 |
| **Yang** | **2019** | 9643 | 9643 | 3586 | 4330 | 0.76 | 0.73 | 0.8 |

Table S1. Supporting data for Forest plot of Hazard Ratio (HR) of post-stroke comorbidities with acupuncture intervention compared with non-acupuncture control. A: acupuncture group, C: control group

| **Study** | **Age** | **HR** | **LCI** | **UCI** |
| --- | --- | --- | --- | --- |
| **chuang2015** | 40-49 | 0.84 | 0.62 | 1.14 |
| **chuang2015** | 50-59 | 0.75 | 0.63 | 0.9 |
| **chuang2015** | 60-69 | 0.85 | 0.75 | 0.95 |
| **chuang2015** | 70-79 | 0.93 | 0.83 | 1.03 |
| **chang2018** | 20-29 | 0.16 | 0.04 | 0.69 |
| **chang2018** | 30-39 | 0.52 | 0.28 | 0.98 |
| **chang2018** | 40-49 | 0.73 | 0.58 | 0.93 |
| **chang2018** | 50-59 | 0.83 | 0.72 | 0.95 |
| **chang2018** | 60-69 | 0.82 | 0.75 | 0.89 |
| **chang2018** | 70-79 | 0.88 | 0.81 | 0.95 |
| **chang2018** | ≥80 | 0.86 | 0.73 | 1 |
| **shih2017** | 50-59 | 0.64 | 0.47 | 0.86 |
| **shih2017** | 60-69 | 0.75 | 0.64 | 0.88 |
| **shih2017** | 70-79 | 0.72 | 0.63 | 0.83 |
| **shih2017** | ≥80 | 0.54 | 0.38 | 0.76 |
| **weng2016** | 20-29 | 0.16 | 0.04 | 0.68 |
| **weng2016** | 30-39 | 0.39 | 0.21 | 0.73 |
| **weng2016** | 40-49 | 0.51 | 0.39 | 0.66 |
| **weng2016** | 50-59 | 0.66 | 0.54 | 0.8 |
| **weng2016** | 60-69 | 0.79 | 0.68 | 0.91 |
| **weng2016** | 70-79 | 0.88 | 0.76 | 1.02 |
| **weng2016** | ≥80 | 0.71 | 0.48 | 1.04 |
| **yang2019** | 30-39 | 0.62 | 0.34 | 1.16 |
| **yang2019** | 40-49 | 0.65 | 0.53 | 0.79 |
| **yang2019** | 50-59 | 0.82 | 0.73 | 0.93 |
| **yang2019** | 60-69 | 0.74 | 0.69 | 0.8 |
| **yang2019** | ≥70 | 0.77 | 0.72 | 0.82 |
| **shih2015** | 30-39 | 0.35 | 0.22 | 0.54 |
| **shih2015** | 40-49 | 0.61 | 0.52 | 0.71 |
| **shih2015** | 50-59 | 0.81 | 0.74 | 0.89 |
| **shih2015** | 60-69 | 0.85 | 0.8 | 0.91 |
| **shih2015** | 70-79 | 0.99 | 0.92 | 1.06 |
| **shih2015** | ≥80 | 1.15 | 0.98 | 1.34 |

Table S2. Supporting data extracted from each study showing HRs with 95% confidence intervals of different age strata on post-stroke comorbidities after acupuncture treatment. (HR: hazard ratio; LCI: lower confidence intervals; UCI: upper confidence intervals)

| **Age** | ***k*** | **HR** | **LCI** | **UCI** | ***I*^2^** | ***p*** |
| --- | --- | --- | --- | --- | --- | --- |
| 20-29 | 2 | 0.1600 | 0.0580 | 0.4368 | 0% | 0.0003 |
| 30-39 | 4 | 0.4362 | 0.3297 | 0.5773 | 0% | <0.0001 |
| 40-49 | 5 | 0.6495 | 0.5666 | 0.7445 | 46.70% | <0.0001 |
| 50-59 | 6 | 0.7819 | 0.7297 | 0.8378 | 23.50% | <0.0001 |
| 60-69 | 6 | 0.8029 | 0.7597 | 0.8485 | 47.50% | <0.0001 |
| 70-79 | 6 | 0.8595 | 0.7777 | 0.9499 | 85.70% | 0.0003 |
| >=80 | 4 | 0.8129 | 0.6075 | 1.0877 | 84.50% | 0.1633 |

Table S3. Meta-analysis results of different age strata on post-stroke comorbidities after acupuncture treatment. (*k*: number of samples; *I*^2^: heterogeneity; *p*: *p*-value)

| **Study** | **Course** | **HR** | **LCI** | **UCI** | **SE** |
| --- | --- | --- | --- | --- | --- |
| **Yang2019** | 0 | 1 | 1 | 1 |  |
| **Yang2019** | 2 | 0.99 | 0.92 | 1.07 | 0.035714286 |
| **Yang2019** | 3 | 0.89 | 0.82 | 0.97 | 0.035714286 |
| **Yang2019** | 4 | 0.89 | 0.8 | 0.98 | 0.045918367 |
| **Yang2019** | 5 | 0.68 | 0.6 | 0.78 | 0.040816327 |
| **Yang2019** | 6 | 0.76 | 0.66 | 0.88 | 0.051020408 |
| **Yang2019** | 7 | 0.67 | 0.57 | 0.79 | 0.051020408 |
| **Yang2019** | 8 | 0.73 | 0.61 | 0.86 | 0.06122449 |
| **Yang2019** | 9 | 0.7 | 0.59 | 0.84 | 0.056122449 |
| **Yang2019** | 10 | 0.63 | 0.51 | 0.79 | 0.06122449 |
| **Yang2019** | 11 | 0.57 | 0.53 | 0.62 | 0.020408163 |
| **Weng 2016** | 0 | 0.77 | 0.66 | 0.9 |  |
| **Weng 2016** | 2 | 1.06 | 0.91 | 1.25 | 0.076530612 |
| **Weng 2016** | 3 | 0.84 | 0.69 | 1.02 | 0.076530612 |
| **Weng 2016** | 4 | 0.75 | 0.59 | 0.95 | 0.081632653 |
| **Weng 2016** | 5 | 0.74 | 0.55 | 0.98 | 0.096938776 |
| **Weng 2016** | 6 | 0.64 | 0.57 | 0.71 | 0.035714286 |
| **Weng 2016** | 7 | 0.69 | 0.63 | 0.74 | 0.030612245 |
| **chuang2015** | 0 | 1 | 1 | 1 |  |
| **chuang2015** | 1 | 0.86 | 0.8 | 0.93 | 0.030612245 |
| **chuang2015** | 2 | 0.79 | 0.73 | 0.86 | 0.030612245 |
| **chuang2015** | 3 | 0.73 | 0.67 | 0.8 | 0.030612245 |
| **chuang2015** | 4 | 0.71 | 0.64 | 0.78 | 0.035714286 |
| **chuang2015** | 5 | 0.68 | 0.61 | 0.76 | 0.035714286 |
| **chuang2015** | 6 | 0.69 | 0.62 | 0.77 | 0.035714286 |
| **chuang2015** | 7 | 0.65 | 0.58 | 0.74 | 0.035714286 |
| **chuang2015** | 8 | 0.66 | 0.58 | 0.75 | 0.040816327 |
| **chuang2015** | 9 | 0.64 | 0.56 | 0.74 | 0.040816327 |
| **chuang2015** | 10 | 0.64 | 0.56 | 0.74 | 0.040816327 |
| **chuang2015** | 11 | 0.64 | 0.55 | 0.74 | 0.045918367 |
| **chuang2015** | 12 | 0.64 | 0.55 | 0.75 | 0.045918367 |
| **chuang2015** | 13 | 0.62 | 0.52 | 0.73 | 0.051020408 |
| **chuang2015** | 14 | 0.63 | 0.53 | 0.77 | 0.051020408 |
| **chuang2015** | 15 | 0.61 | 0.51 | 0.73 | 0.051020408 |
| **Chang 2018** | 0 | 1 | 1 | 1 |  |
| **Chang 2018** | 2 | 0.98 | 0.91 | 1.07 | 0.035714286 |
| **Chang 2018** | 3 | 0.99 | 0.9 | 1.09 | 0.045918367 |
| **Chang 2018** | 4 | 0.88 | 0.78 | 0.99 | 0.051020408 |
| **Chang 2018** | 5 | 0.84 | 0.73 | 0.96 | 0.056122449 |
| **Chang 2018** | 6 | 0.77 | 0.73 | 0.82 | 0.020408163 |
| **shih2015** | 0 | 1 | 1 | 1 |  |
| **shih2015** | 1 | 1.03 | 0.97 | 1.09 | 0.030612245 |
| **shih2015** | 2 | 0.91 | 0.84 | 0.98 | 0.035714286 |
| **shih2015** | 3 | 0.89 | 0.81 | 0.98 | 0.040816327 |
| **shih2015** | 4 | 0.85 | 0.76 | 0.95 | 0.045918367 |
| **shih2015** | 5 | 0.77 | 0.73 | 0.81 | 0.020408163 |

Table S4. Supporting data extracted from each study showing HRs with 95% confidence intervals of various acupuncture courses on post-stroke comorbidities after acupuncture treatment.

| **Course number** | ***k*** | **HR** | **LCI** | **UCI** | ***I*^2^** | ***p*** |
| --- | --- | --- | --- | --- | --- | --- |
| 0 | - | 1 | 1 | 1 | - | - |
| 1 | 2 | 0.9427 | 0.79 | 1.125 | 92.70% | 0.513 |
| 2 | 5 | 0.9343 | 0.851 | 1.0256 | 82.10% | 0.1533 |
| 3 | 5 | 0.865 | 0.7757 | 0.9646 | 82.30% | 0.0091 |
| 4 | 5 | 0.8186 | 0.742 | 0.9031 | 69.10% | < 0.0001 |
| 5 | 5 | 0.7411 | 0.6853 | 0.8015 | 54.50% | < 0.0001 |
| 6 | 4 | 0.7151 | 0.6524 | 0.7839 | 70.20% | < 0.0001 |
| 7 | 3 | 0.6765 | 0.6358 | 0.7198 | 0.00% | < 0.0001 |
| 8 | 2 | 0.6843 | 0.6174 | 0.7585 | 0.00% | < 0.0001 |
| 9 | 2 | 0.6624 | 0.5937 | 0.739 | 0.00% | < 0.0001 |
| 10 | 2 | 0.6371 | 0.5664 | 0.7166 | 0.00% | < 0.0001 |
| 11 | 2 | 0.5933 | 0.5326 | 0.661 | 45.40% | < 0.0001 |

Table S5. Supporting data for Meta-analysis results of various acupuncture courses on post-stroke comorbidities after acupuncture treatment. (*k*: number of samples; *I*^2^: heterogeneity; *p*: *p*-value)

**Subgroup meta-analysis of post-stroke depression**

After studies screening and selection, eight articles were included in our systematic review. Moreover, two included articles are studies of post-stroke depression. The association of acupuncture and post-stroke depression were inconsistent in two studies. Tseng (2017) indicated the reduced risk of post-stroke depression with HR of 0.48 (95% CI: 0.39-0.58) and 0.72 (95% CI: 0.61-0.84) for patients who received frequent and infrequent acupuncture treatments, respectively. Conversely, Lu (2017) reported a HR of 1.04 (95% CI: 0.84-1.29). As a result, we conduct a meta-analysis on those two studies as a supplementation. After meta-analysis with random effects model, the result showed a HR of 0.70 (95% CI: 0.47-1.05) favors acupuncture but without significant difference.


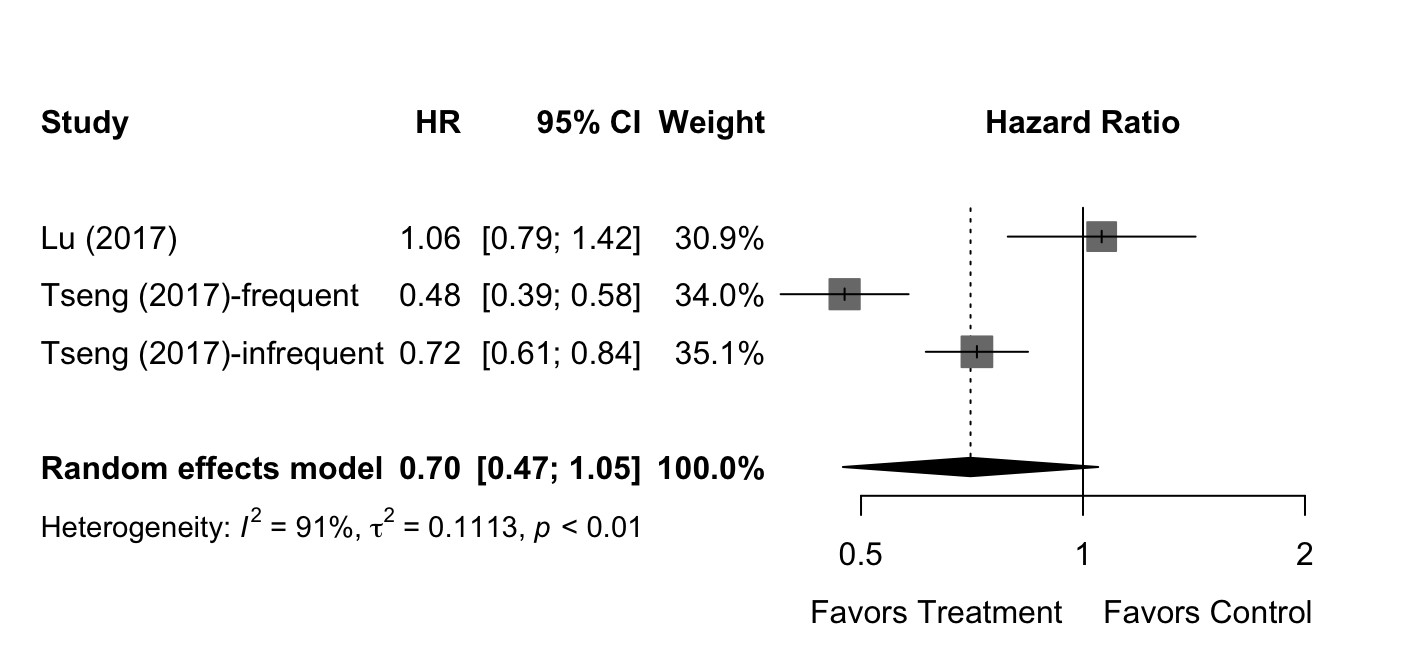


Figure S1. Forest plot of Hazard Ratio (HR) of post-stroke depression with acupuncture intervention compared with non-acupuncture control.
