# Supplementary figures and images for: Efficacy of Acupuncture Treatment for Incidence of Poststroke Comorbidities: A Systematic Review and Meta-Analysis of Nationalized Cohort Studies
Source: Evid Based Complement Alternat Med. 2022 Feb 1;2022:3919866. doi: 10.1155/2022/3919866 (PMC8825287; doi:10.1155/2022/3919866)

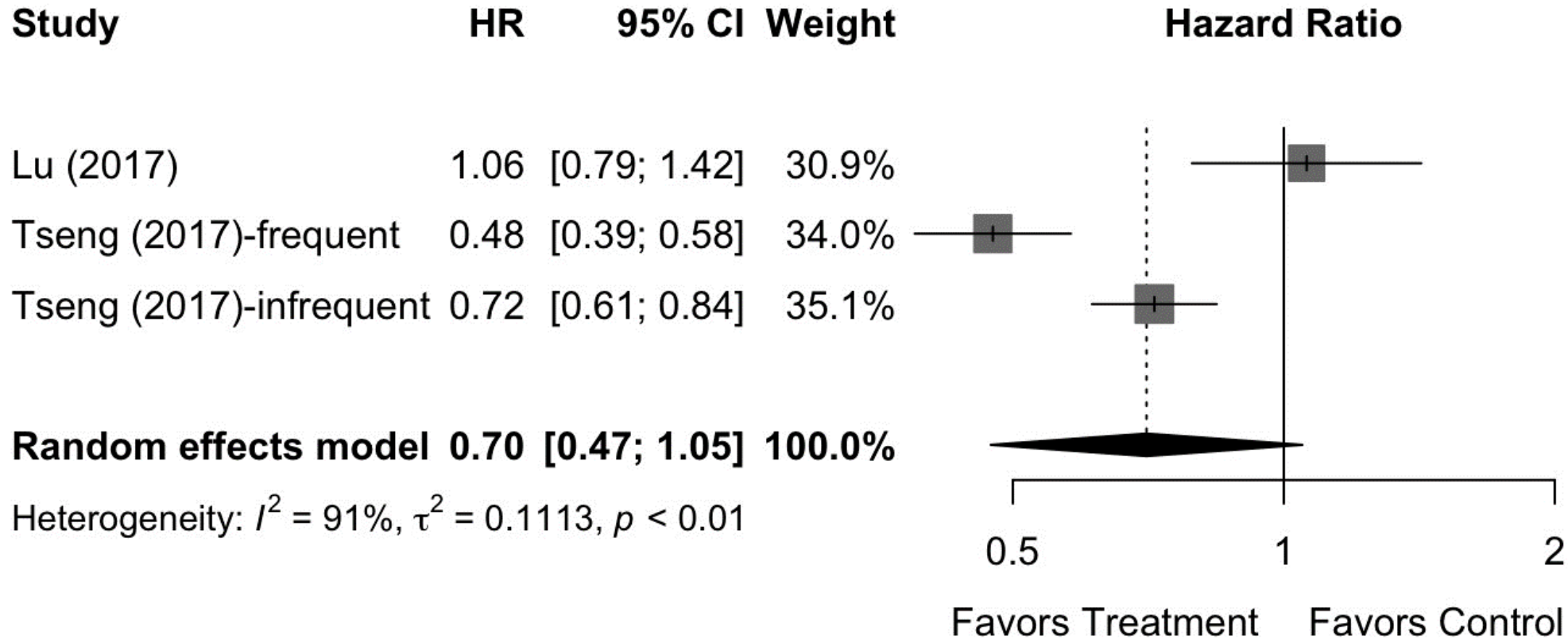

Supplement: Supplementary Materials — Table S1. Supporting data for Forest plot of Hazard Ratio (HR) of post-stroke comorbidities with acupuncture intervention compared with non-acupuncture control. A: acupuncture group, C: control group. Table S2. Supporting data extracted from each study showing HRs with 95% confidence intervals of different age strata on post-stroke comorbidities after acupuncture treatment. (HR: hazard ratio; LCI: lower confidence intervals; UCI: upper confidence intervals). Table S3. Meta-analysis results of different age strata on post-stroke comorbidities after acupuncture treatment. (k: number of samples; I2: heterogeneity; p: p-value). Table S4. Supporting data extracted from each study showing HRs with 95% confidence intervals of various acupuncture courses on post-stroke comorbidities after acupuncture treatment. Table S5. Supporting data for Meta-analysis results of various acupuncture courses on post-stroke comorbidities after acupuncture treatment. (k: number of samples; I2: heterogeneity; p: p-value). Figure S1. Forest plot of Hazard Ratio (HR) of post-stroke depression with acupuncture intervention compared with non-acupuncture control. [file 3919866.f1.zip › Figure 6 Supplementation (3).pdf]
